# Exploring the Suitability of High-Performance Thin-Layer Chromatography for the Analysis of Drug Admixtures in Total Parenteral Nutrition Using Caffeine, Ibuprofen and Midazolam as Model Drugs

**DOI:** 10.3390/molecules31162904

**Published:** 2026-08-20

**Authors:** K. M. Yasif Kayes Sikdar, Md Khairul Islam, Tomislav Sostaric, Lee Yong Lim, Marcus Femia, Cornelia Locher

**Affiliations:** 1 Department of Pharmacy and Centre for Optimisation of Medicines, School of Health and Clinical Sciences, University of Western Australia, Perth, WA 6009, Australia; kmyasifkayes.sikdar@uwa.edu.au (K.M.Y.K.S.); khairul.islam@uwa.edu.au (M.K.I.); tom@chromatechscientific.com (T.S.); lee.lim@uwa.edu.au (L.Y.L.); 2 Institute for Paediatric Perioperative Excellence, University of Western Australia, Perth, WA 6009, Australia; 3 Pharmacy Department, Women and Newborn Health Service, King Edward Memorial Hospital, Subiaco, WA 6008, Australia; marcus.femia@health.wa.gov.au

**Keywords:** total parenteral nutrition (TPN), drug admixing, chemical compatibility, HPTLC

## Abstract

Total parenteral nutrition (TPN) is a nutritional therapy in children when oral or enteral feeding is not possible, fails to meet nutritional requirements or is contraindicated due to underlying clinical conditions. There are many cases where children who are given one or more than one intravenous medication also need TPN. In order to minimise vein access, these medications are commonly administered via a Y-site IV connection where they admix with TPN, implying a need for compatibility studies before admixing the medication into the nutritional fluid. In this study, previously developed and validated HPTLC methods were verified for their suitability to evaluate the concentrations of three commonly used medications in neonatal intensive care units (NICUs), namely caffeine citrate, ibuprofen arginine, and midazolam hydrochloride. Silica gel 60 F254 HPTLC plates were used as the stationary phase, and acetone: toluene: chloroform (4:3:3, *v*/*v*/*v*), toluene: methanol: ethyl acetate: glacial acetic acid (5.5:3.2:1:0.3, *v*/*v*/*v*/*v*), and cyclohexane: toluene: diethylamine (7:1.5:1.5, *v*/*v/v*) with one drop of 25% ammonia solution were used as mobile phases. The compatibility of the three drugs in their respective TPN admixtures was then assessed at various time points over a 24 h period using these HPTLC methods. Each drug admixed in TPN was directly applied onto the HPTLC plates without further sample pre-treatment. Paired Two-One-Sided Test (TOST) equivalent testing revealed that the concentrations of the selected drugs in TPN were unchanged over the monitoring period (*p* > 0.05), confirming chemical compatibility. These findings were in line with previous studies that concluded compatibility and reported similar R_F_ and LOD/LOQ values (1.95/5.90 ng/band for caffeine, 252.22/764.30 ng/band for ibuprofen and 10.82/32.78 ng/band for midazolam; *R*^2^ ≥ 0.99). As no additional sample pre-treatment was required, HPTLC-based compatibility studies of drug–TPN admixtures may simplify the analytical workflow, making them a cost-effective and time-efficient approach to compatibility testing.

## 1. Introduction

Total parenteral nutrition (TPN) involves the intravenous administration of macronutrients such as dextrose, proteins, and lipids alongside micronutrients, including electrolytes, multivitamins, and trace elements [1,2,3]. TPN is used when oral or enteral feeding is not an option, inadequate, or inadvisable. Because it provides essential nutrients to prevent or correct nutritional deficiencies and supports normal growth and development, TPN may be lifesaving for children with severe nutritional deficiencies [4].

In clinical practice, TPN may need to be co-administered with other IV medications. When vein access is limited or fluid overload is a concern, such as in very young patients, these drugs might be given concurrently with TPN via a Y-site IV connection. There are a number of intravenous drugs that can be administered via Y-site connectors simultaneously at high concentrations and low flow rates in neonatal intensive care units (NICUs) [5,6,7]. Given the complex chemical composition of TPN, such combinations might, however, lead to physical and/or chemical interactions that make the simultaneous delivery of a drug alongside TPN impossible [5,6,7,8,9,10]. Compatibility studies are therefore required, which typically involve not only monitoring for potential physicochemical interactions, seen, for example, in a change in pH, the formation of turbidity or colour development over a period of time, but also chemical interactions that would manifest in reduced drug concentration on contact with TPN constituents. While observations of potential colour change and turbidity, as well as the monitoring of pH changes, are simple to perform and therefore routinely carried out in drug–drug and also drug–TPN interaction analyses [7,9,11], the absence of such interactions does not necessarily imply full compatibility. Interactions between drug and TPN components might take place, but not result in measurable changes to pH and colour or lead to the formation of a visible precipitate. Moreover, drugs might bind to TPN components, such as lipids, forming complexes that reduce the unbound drug concentration delivered to the patient, resulting in sub-therapeutic doses. The analysis of such types of interaction is more challenging given the multi-component nature of the TPN mixture, featuring constituents with a diverse range of chemical properties. High-Performance Liquid Chromatography (HPLC), for example, despite being the method of choice for most compatibility studies, might require extensive pre-analysis extraction steps to remove lipid components that otherwise might hamper the analysis [12].

In light of these challenges, this study explored the suitability of High-Performance Thin-Layer Chromatography (HPTLC) as an alternative analytical tool to assess the chemical compatibility of drugs in TPN admixtures. The rationale for this approach is the demonstrated ability of silica gel plates, the stationary phase commonly used in HPTLC analyses, to support the in situ separation of lipid components without the need for pre-analysis liquid–liquid or SPE-cartridge-based extractions [13], possibly resulting in much less complex analysis protocols for drug–TPN admixtures. Moreover, given HPTLC’s high throughput capability, with the option of a concurrent analysis of up to 20 samples using minimal solvent and reagent input [14], this analytical approach might also be more efficient and greener than conventional techniques [15,16,17].

**Figure 1:**
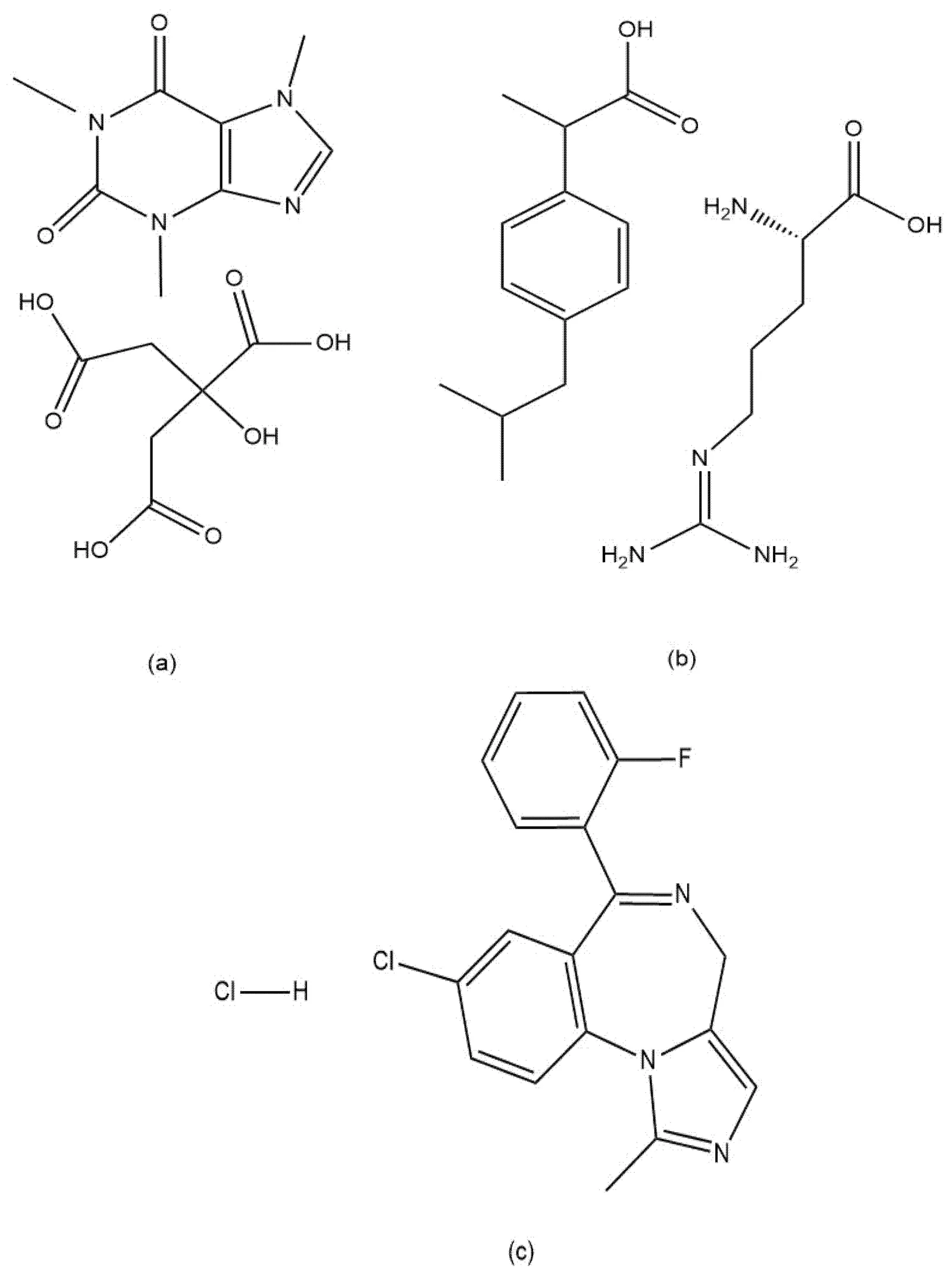
Chemical structures of (**a**) caffeine citrate (CAF), (**b**) ibuprofen arginine (IBP), and (**c**) midazolam hydrochloride (MDZ).

The TPN selected for this study contained amino acids, glucose, electrolytes, paediatric trace elements and heparin sodium combined with a lipid emulsion containing refined soya oil, medium chain triglycerides, refined olive oil, fish oil and fat-soluble vitamins. The three model drugs chosen for this study were caffeine citrate (CAF), ibuprofen arginine (IBP), and midazolam hydrochloride (MDZ) (Figure 1), as previously developed and validated HPTLC methods for these drugs were available.

Treatment for apnoea among preterm infants involves the administration of caffeine, which is recognized as one of the most beneficial drugs in treating this condition, owing to its broad therapeutic window and long elimination half-life [18,19,20,21]. According to international treatment guidelines, caffeine citrate (CAF), which is used in intravenous (IV) administration routes, is considered physiochemically compatible with parenteral nutrition (PN) components but incompatible with lipid emulsions. These guidelines are based on HPLC-derived data, which require complex sample pre-treatment involving centrifugation of the samples and subsequent evaporation of supernatants before analysis [22,23,24].

Patent ductus arteriosus (PDA) is another serious disorder, particularly affecting low-weight infants. In many neonatal hospital units, ibuprofen is commonly the medication of choice in the treatment of this condition because of its lower risk of renal and gastrointestinal complications compared to commonly used NSAIDs like indomethacin [25,26,27]. However, despite its popularity, studies concerning its compatibility with TPN are rare, although one study found the drug to be physically and chemically compatible for up to 4 h with TPN-relevant components such as 10% glucose, starter PN, standard preterm PN, low-carbohydrate PN, and a lipid emulsion admixture supplemented with vitamins [12]. Existing studies used HPLC as primary analytical tool for this task, but the analytical approach required centrifugation as a sample pre-treatment [12,28].

Midazolam hydrochloride (MDZ) is commonly used as a procedure sedative, anaesthetic inducer and maintenance drug in the treatment of chronic liver disease, as well as for sedation therapy in intensive care units, because of its relatively short duration of action and good safety profile [29,30,31]. From previous studies, it appears that the most common analytical tool for confirming the chemical compatibility of MDZ admixtures in a PN solution is HPLC [29,32,33,34]. However, this approach requires sample filtration with 0.45-micron HPLC nylon filters prior to analysis. No previous studies analysing the compatibility of IV midazolam admixed with TPN have been reported to date. In addition, the local Y-site Compatibility in Neonates Guideline currently lacks compatibility data for midazolam admixed with lipid emulsions [3].

## 2. Results and Discussion

### 2.1. Chromatographic Condition Selection

**Figure 2:**
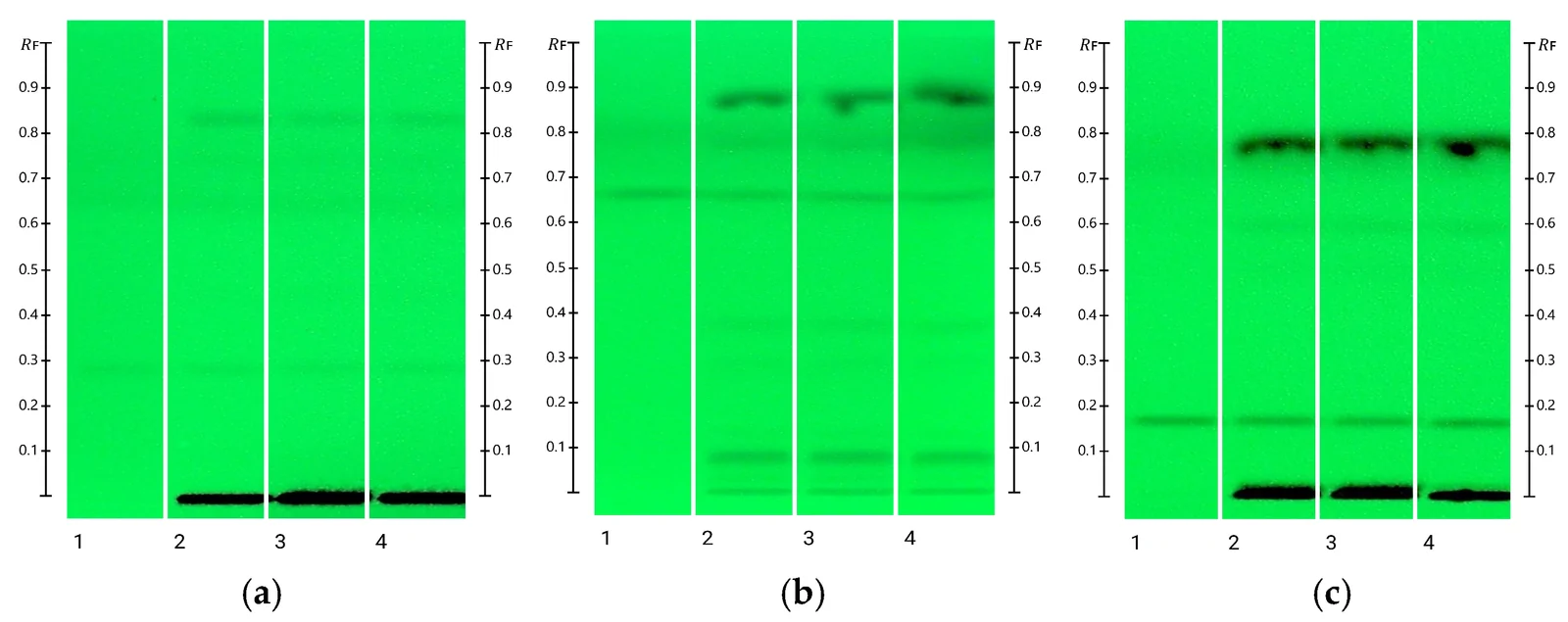
HPTLC fingerprints of (**a**) Track 1: Caffeine standard (R_F_: 0.27); Tracks 2, 3, and 4: Caffeine citrate (CAF) admixed in TPN; (**b**) Track 1: Ibuprofen standard (R_F_: 0.67); Tracks 2, 3, and 4: Ibuprofen arginine (IBP) admixed in TPN; (**c**) Track 1: Midazolam standard (R_F_: 0.17); Tracks 2, 3, and 4: Midazolam hydrochloride (MDZ) admixed in TPN.

Three previously reported mobile phases—acetone: toluene: chloroform (4:3:3, *v*/*v*/*v*), toluene: methanol: ethyl acetate: glacial acetic acid (5.5:3.2:1:0.3, *v*/*v*/*v*/*v*), and cyclohexane: toluene: diethylamine (7:1.5:1.5, *v*/*v/v*) with one drop of 25% ammonia solution—were selected for analysing CAF, IBP, and MDZ in TPN. After chromatographic separation, the three drugs exhibited distinct R_F_ values at 0.27 ± 0.03, 0.67 ± 0.02 and 0.16 ± 0.03, respectively (Figure 2), consistent with previously reported data [35,36,37] and well separated from other TPN constituents. The corresponding HPTLC fingerprints were recorded at 254 nm and processed using visionCATS 4.0 (CAMAG, Muttenz, Switzerland) software. The absorption maxima (λ_max_) were found to be at 275 nm for caffeine, 271 nm for ibuprofen and 229 for midazolam, which were also in line with data reported in previous studies (Table 1) (Figure S1) [35,36,37]. Quantification of all three drugs showed high reproducibility when peak height was plotted against concentration.

### 2.2. Method Verification

#### 2.2.1. Specificity

The specificity of the HPTLC methods was verified by comparing the R_F_ values of the reference standards with those of the corresponding analytes in the TPN admixtures. After chromatographic separation, the R_F_ values obtained for CAF, IBP, and MDZ in the TPN matrix were 0.27 ± 0.03, 0.67 ± 0.02, and 0.16 ± 0.03, respectively, which were identical to those of the corresponding standards dissolved in methanol. Moreover, there were no bands that interfered with the analytes from any of the TPN ingredients, which confirmed the specificity of the HPTLC procedure for analysing caffeine, ibuprofen, and midazolam as TPN admixtures (Figure 2).

#### 2.2.2. Linearity

Calibration curves constructed for caffeine (20–100 µg/mL), ibuprofen (1000–5000 µg/mL), and midazolam (50–175 µg/mL) demonstrated excellent linearity over the investigated concentration ranges. The coefficients of determination (*R*^2^) were found to be greater than 0.99 for all three analytes, confirming the strong linear relationship between peak height and analyte concentration (Table 1).

#### 2.2.3. Sensitivity

The sensitivity of the HPTLC methods was verified by calculating the limits of detection (LOD) and quantification (LOQ) from the corresponding standard curves using the mean slope and y-intercepts obtained from linear regression analyses. Results revealed that the respective LOD/LOQ values for the three drugs (Table 1)—namely, 1.95/5.90 ng/band for caffeine, 252.22/764.30 ng/band for ibuprofen, and 10.82/32.78 ng/band for midazolam—were comparable to those of previously developed and validated HPTLC methods (caffeine: 2.42/7.34 ng/band; ibuprofen: 141.33/428.28 ng/spot; and midazolam: 30/70 ng/band), confirming that the HPTLC methods verified in this study had similar sensitivity to earlier reports [35,36,37].

#### 2.2.4. Accuracy and Precision

The accuracy of the HPTLC methods was evaluated based on the average % recovery of each analyte. As summarised in Table 1, the recovery values for caffeine, ibuprofen, and midazolam were within the acceptable limits of the International Conference on Harmonisation (ICH) guidelines, confirming the accuracy of the previously developed HPTLC methods [38]. Their intra- and inter-day precision were also assessed based on their respective % relative standard deviation (%RSD). All %RSD values were less than 5%, demonstrating excellent precision according to the limits set out in the ICH guidelines [38].

#### 2.2.5. Repeatability

All drugs were tested in five replicates using their respective HPTLC methods. Method repeatability was assessed based on %RSD, and all values were within the acceptable limits specified by the ICH guidelines. The results are summarised in Table 1.

### 2.3. Chemical Compatibility Assessment

**Figure 3:**
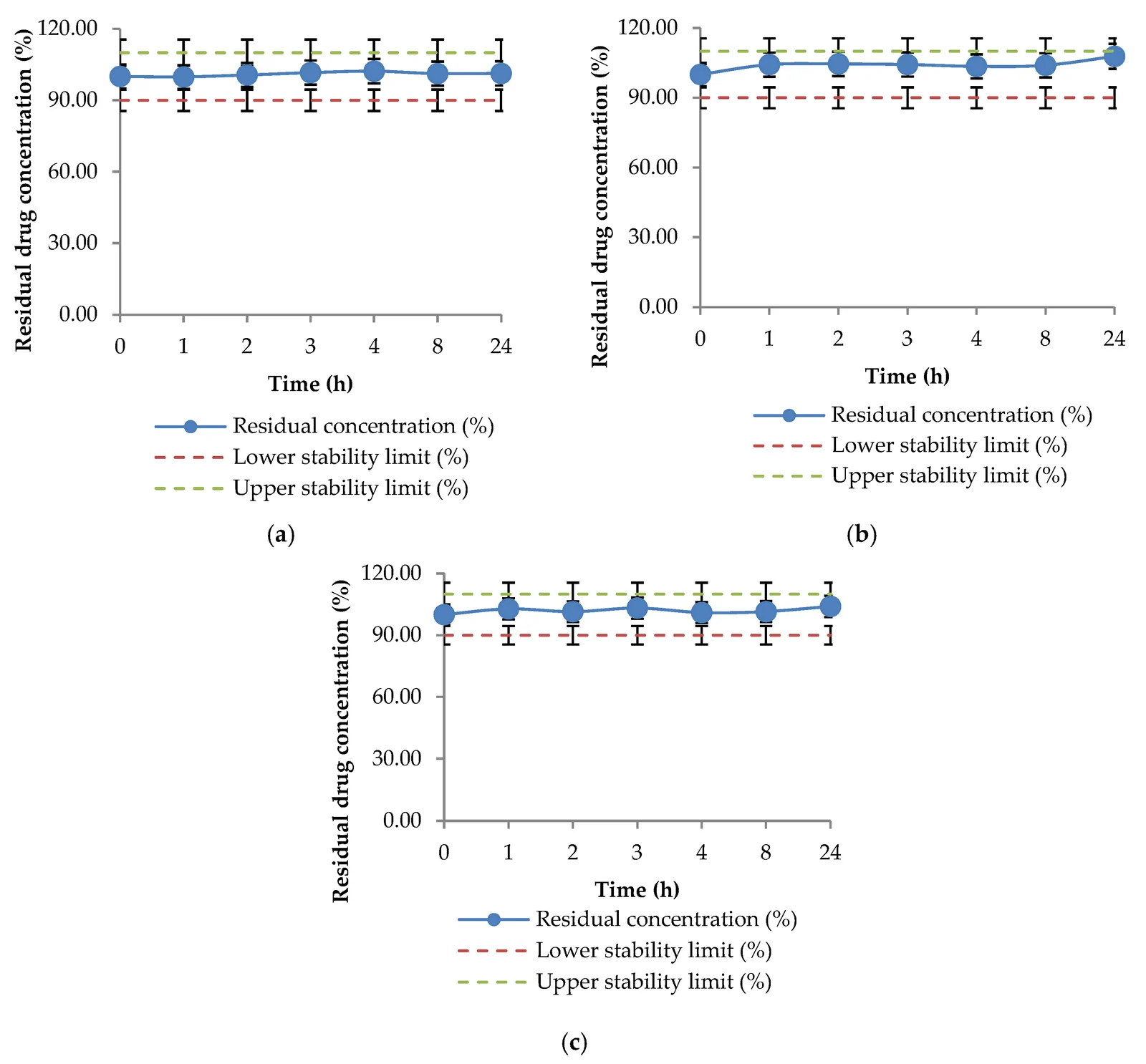
Residual drug concentrations of (**a**) residual drug concentration of CAF, (**b**) residual drug concentration of IBP and (**c**) residual drug concentration of MDZ in TPN admixture at different time points at room temperature. Drug concentrations are expressed as mean ± SD (*n* = 3). Lower and higher stability limit ranges: 90–110% of initial concentration.

Thew chemical compatibility of the drugs admixed with TPN was monitored over a 24 h time period with sampling at 1, 2, 3, 4, 8, and 24 h. The residual drug concentrations of admixed CAF, IBP and MDZ in TPN were within the predefined limit (90–110% of the initial concentration) throughout the 24 h period and are shown in Figure 3a–c. The collected data reveal that the drugs’ concentrations in TPN over the 24 h monitoring period were not significantly different from their theoretical concentrations. A paired Two-One-Sided Test (TOST) for equivalence testing was performed for each drug admixed in TPN. All drug concentrations at different time points for CAF, IBP, and MDZ were statistically equivalent (*p* > 0.05) to the baseline (0 h) concentration, except for 24 h for IBU, which showed greater variability among replicates (Table S1), indicating that the respective drug contents remained consistent throughout the study period.

Although a previous study concluded that CAF is not physically compatible with lipid emulsions [39], the findings of the present study demonstrated that CAF admixed with TPN, which also included lipid constituents, is chemically compatible. Moreover, the findings of this study are in line with previous work using HPLC analysis after sample pre-treatment [22,24], which demonstrated that CAF concentrations remained chemically stable for up to 24 h following admixture with PN solution. Despite the known physical incompatibility of CAF with lipid admixtures, the developed HPTLC method can successfully evaluate the chemical stability of CAF in TPN admixtures without requiring any sample extraction or pre-treatment prior to analysis. This further highlights the advantages of the developed HPTLC method as a simple, rapid and cost-effective approach in assessing the chemical stability of CAF in the TPN formulation system.

Similarly, in a previous compatibility investigation, IBP was already found to be compatible with TPN over a 4 h contact period where HPLC was used to analyse the drug in solution after sample pre-treatment (centrifugation) [12]. However, this HPTLC-based study can be considered more convenient, as multiple samples collected over a 24 h period were analysed concurrently on a single plate with only 10 mL of solvent input and no requirement for any pre-analysis treatment.

Using a HPLC method [32], previous work also demonstrated that MDZ remains stable in PN solutions for up to 5 h, which is consistent with the findings of the present HPTLC study, which offers prolonged stability data up to 24 h. However, local neonatal Y-site compatibility guidelines do not provide compatibility data for midazolam admixed with lipid emulsions [3]. Based on the current study, which used a TPN solution, it can be concluded that there is chemical compatibility with lipids.

In this study, HPTLC analysis has been shown to offer a simple, rapid, and cost-effective approach for evaluating the chemical compatibility of drugs in complex TPN admixtures. Given the chemical differences in the three model drugs used, it can also be concluded that the analytical approach is very versatile and suited to drugs that differ widely in their polarity, ionisation characteristics, and water solubility (Table 2) without compromising analytical performance [40,41]. The broad application potential suggests that HPTLC analysis can conveniently be used in high-throughput compatibility studies of various drug–TPN admixtures, including those commonly used in NICU settings. Although three chemically diverse drugs were used as model drugs in this study, additional studies with more drugs are required to confirm the suitability of HPTLC methods more broadly for drug–TPN admixture compatibility studies.

## 3. Materials and Methods

### 3.1. Chemicals, Materials, and Commercial Samples

Caffeine was purchased from Sigma (Sydney, NSW, Australia), ibuprofen was sourced from Professional Compounding Chemists of Australia Pty. Ltd. (Matraville, NSW, Australia) and midazolam was bought from Cambrex Profarmaco Milano S.r.l. (Paullo, Milan, Italy). All solvents used in this study were of analytical grade. Acetone, chloroform, methanol and diethylamine were obtained from Merck KGaA (Darmstadt, Germany); cyclohexane, glacial acetic acid, ethyl acetate, t-butanol, and n-propanol were purchased from Chem Supply Pty. Ltd. (Gillman, SA, Australia). Toluene was obtained from Honeywell (Muskegon, MI, USA), while n-hexane was sourced from RCI Labscan Ltd. (Pathumwan, Bangkok, Thailand). We used caffeine citrate injection BP (50 mg/5 mL; prepared by Perth Children’s Hospital, Perth, WA, Australia), sodium chloride 0.9% injection BP (Cremorne, VIC, Australia), midazolam hydrochloride injection (5 mg/5 mL; Cheprla Pharm, Greifswald, Germany), ibuprofen arginine injection (800 mg/8 mL, Phebra, NSW, Australia), PN base solution and a pre-filled 25 mL syringe of SMOF 20% fat emulsion with vitamins (SMOFlipids) (Table 3) containing soya oil (30%), medium chain triglycerides (30%), refined olive oil (25%), and fish oil (15%) (prepared and provided by the Pharmacy Department of King Edward Memorial Hospital (KEMH), Subiaco, WA, Australia). Deionised water, produced by a reverse osmosis system (PSI Water Filters Australia, Launceston, TAS, Australia), was used throughout the experiment. Silica gel 60 F254 HPTLC glass plates (20 cm × 10 cm) were obtained from Merck KGaA (Darmstadt, Germany).

### 3.2. Sample Preparation and Analysis

CAF, IBP and MDZ admixed in TPN solutions were prepared following the local NICU drug administration protocols [3]. Drug concentrations were selected based on the standard intravenous infusion doses prescribed for a 2 kg patient. Drug–TPN admixtures containing the desired concentrations of CAF (60 µg/mL), IBP (2500 µg/mL), and MDZ (150 µg/mL) were prepared by mixing 10.4 mL lipid emulsion into the PN base solution, after which the respective drug solutions were added and the mixture was then made up to 25 mL using the PN base solution. All the solutions were mixed and sonicated for 5 min and then assessed through the naked eye for the presence of any undissolved particles. Following preparation, each drug admixture was sampled immediately after sonication, as well as after 1, 2, 3, 4, 8, and 24 h storage at room temperature. Collected samples were stored immediately at −20 °C before concurrent analysis on a single plate using the respective verified HPTLC methods without any further pre-analysis treatment or extraction. The HPTLC replicate analyses were performed to evaluate the chemical stability of each drug in the TPN admixture. A TOST-equivalent test was performed to evaluate whether the concentrations of CAF, IBP and MDZ in the TPN admixtures changed significantly over the sampling period.

### 3.3. Mobile Phase Selection and Stock Solution Preparation

To analyse CAF, IBP, and MDZ admixed in TPN, mobile phases from previously developed and validated HPTLC methods were trialled to identify the most suitable chromatographic conditions.

The mobile phase acetone: toluene: chloroform (4:3:3, *v*/*v*/*v*) was previously reported for the analysis of caffeine in human saliva, mate beer and soft drinks using HPTLC [35,42]. For the analysis of ibuprofen, the mobile phases t-butanol: ethyl acetate: glacial acetic acid: water (7:4:2:2, *v*/*v/v*), n-hexane: ethyl acetate: anhydrous acetic acid (75:25:2, *v*/*v*/*v*), and toluene: methanol: ethyl acetate: glacial acetic acid (5.5:3.2:1:0.3, *v*/*v*/*v*/*v*) were evaluated. These mobile phases were previously reported for the determination of ibuprofen in tablet dosage forms and plasma samples [36,43,44]. The mobile phases cyclohexane: toluene: diethylamine (7:1.5:1.5, *v*/*v/v*) with one drop 25% ammonia solution and ethyl acetate: *n*-propanol: water: glacial acetic acid (60:24:9:3, *v*/*v*/*v*/*v),* which were used previously for assessing the compatibility and stability of MDZ ternary admixtures and for the forensic analysis of MDZ in urine, respectively [17,37], were evaluated for the analysis of MDZ in TPN.

Individual caffeine, ibuprofen, and midazolam stock solutions (1 mg/mL) were prepared by weighing 10 mg of each drug into 10 mL volumetric flasks and dissolving them in 10 mL of methanol using 5 min of sonication (Ultrasonicator, Unisonics Pty. Ltd., Sydney, NSW, Australia). Working solutions of caffeine and midazolam (25 µg/mL) were prepared using the respective stock solutions while the stock solution of ibuprofen (1 mg/mL) itself was used as a working solution. All stock and working solutions were kept at −20 °C prior to analysis.

### 3.4. HPTLC Instrumentation, Sample Application and Development

Calibration curves of caffeine, ibuprofen and midazolam were established over concentration ranges from 20–100 µg/mL, 1000–5000 µg/mL and 50–175 µg/mL, respectively. The corresponding application volumes listed in Table 4 were applied on the HPTLC plates by using a semi-automated HPTLC applicator (Linomat 5, CAMAG, Muttenz, Switzerland). In Table 4, the application parameters for each drug, including the application rate, band width and distance between bands, are summarised. Nitrogen gas was used as the spraying gas, which was maintained at 600 kPa throughout the application.

The instrumental and operating conditions for the analysis of the three drugs, which are summarised in Table 4, were adopted from previously published methods [35,36,37].

In brief, chromatographic separation of caffeine, ibuprofen and midazolam was performed using an automated development chamber (ADC2, CAMAG, Muttenz, Switzerland), which was saturated and activated at a relative humidity of 33% prior to development. Silica gel 60 F254 HPTLC plates (20 × 10 cm) were used as the stationary phase throughout the study. Following chamber saturation with the corresponding mobile phase, HPTLC plates were pre-conditioned for 5 min using a saturation pad and then developed to the desired migration distance. After development, the plates were dried for 5 min before evaluation. For this, analyses were performed at 254 nm using the TLC Visualizer 2 (CAMAG, Muttenz, Switzerland). Developed bands were then scanned with the TLC Scanner 4 (CAMAG, Muttenz, Switzerland) using a scan speed of 20 mm s^−1^, a step size of 100 µm/step, and a slit size of 5.0 × 0.2 mm^2^. All HPTLC modules were operated, and the chromatographic data were evaluated by the HPTLC visionCATS 4.0 software (CAMAG, Muttenz, Switzerland).

### 3.5. Verification of HPTLC Methods

According to the Analytical Procedures and Methods Validation for Drugs and Biologics Guidance for Industry published by the US Food and Drug Administration (US FDA), the suitability of a validated analytical method should be verified under actual conditions of use. The verification protocol must include various validation parameters (e.g., specificity, LOD, LOQ, precision and accuracy) depending on the extent of the verification process [45]. In this study, the HPTLC methods for analysing caffeine, ibuprofen and midazolam were verified comprehensively by assessing the specificity, linearity, sensitivity, precision, accuracy and repeatability of previously validated methods according to the International Conference on Harmonisation (ICH) guidelines Q2 (R2) [38]. Although the HPTLC methods were previously validated, they were validated for analysing molecules in simpler matrices such as tablet dosage forms or in other complex matrices such as saliva and urine; therefore, verification of the selected drugs admixed in TPN was required because of potential interference from this matrix’s complex composition [35,36,37].

#### 3.5.1. Specificity

The specificity is the extent to which an analytical procedure can accurately identify and quantify the analyte of interest in the presence of other substances that may be present within the sample matrix. The specificity of the HPTLC methods to analyse caffeine, ibuprofen, and midazolam was assessed by comparing the R_F_ values of the reference standards with those obtained for the corresponding analytes in TPN as the sample matrix at the same concentration level. Moreover, peak purities of the respective drug bands in the admixtures were evaluated by comparing the absorption spectra of sample bands with those of the corresponding reference standard bands.

#### 3.5.2. Linearity

Linearity defines the ability of the method to yield results that are directly proportional to the concentration of the compound being analysed. In this study, linearity was evaluated by using five-point calibration curves for caffeine (20–100 µg/mL) and ibuprofen (1000–5000 µg/mL) and a six-point calibration curve for midazolam (50–175 µg/mL). The selected concentration ranges were verified for linearity based on previously reported methods [35,36,37]. Replicate analyses were performed for each drug and the linearity for each drug was assessed using the coefficient of determination (*R*^2^), slope (m), y-intercept, and standard deviation (SD). All calculations were carried out using Microsoft Excel^®^.

#### 3.5.3. Sensitivity

The sensitivity of an analytical method refers to its ability to identify and quantify the smallest amount of analyte in a sample solution. The sensitivity of the methods was assessed in terms of limit of detection (LOD) and limit of quantification (LOQ), calculated according to ICH guidelines using the following equations [38]:

LOD = 3.3 × the mean standard deviation of the regression lines (σ)/average slope of the calibration curves (S)

LOQ = 10 × the mean standard deviation of the regression lines (σ)/average slope of the calibration curves (S)

#### 3.5.4. Accuracy

The accuracy of an analytical method relates to the closeness of the observed outcomes to the expected values of the analyte. To determine the accuracy of the methods, three different amounts of caffeine (20, 40 and 60 ng/band), ibuprofen (2000, 3000 and 4000 ng/band), and midazolam (75, 100, and 125 ng/band) were quantified. The recovery study was conducted in triplicate, and the results were expressed as the % recovery and %RSD of the analysed caffeine, ibuprofen and midazolam standards.

#### 3.5.5. Precision

Precision refers to the extent to which measurements within a dataset agree with one another. It is measured in terms of intra-day and inter-day precision, which may include differences between measurements caused by changes in the analyst, instrument, or laboratory. In this study, the precision of the HPTLC methods for analysing caffeine, ibuprofen, and midazolam was evaluated in terms of intra-day and inter-day precision. The results were expressed as percent relative standard deviation (%RSD) which should be less than 5% to indicate a precise method.

#### 3.5.6. Repeatability

In method validation, repeatability is concerned with the precision of the method through the degree of agreement between different test results conducted under similar conditions. Repeatability of the methods in this study was evaluated by analysing a specific sample concentration five times: 40 ng/band, 2000 ng/band, and 100 ng/band for caffeine, ibuprofen, and midazolam, respectively. The results were expressed as %RSD which should be below 5% according to ICH guidelines [38].

## 4. Conclusions

In this study, previously developed and validated HPTLC methods were verified and applied to the analysis of three commonly used NICU drugs—caffeine citrate, ibuprofen arginine, and midazolam hydrochloride—admixed in TPN for a 24 h time period [35,36,37]. Given their chemical differences, the selected drugs served as model drugs to evaluate the suitability of HPTLC analysis for compatibility studies of a broad range of drug admixtures in TPN. The concentrations of the model drugs in their respective TPN admixtures remained stable over a 24 h time period. A paired TOST-equivalent test confirmed that all drug concentrations at different time points were statistically equivalent (*p* > 0.05) to the baseline (0 h) concentration. These findings are consistent with previously published data that demonstrated the chemical compatibility of CAF and MDZ in PN and IBP in TPN solutions. Moreover, they highlight the advantages of HPTLC over other analytical techniques, particularly in terms of eliminating sample pre-treatment steps and providing the option to analyse collected samples in a single run with minimal solvent input, thereby reducing overall analysis time and costs. The results highlight the efficiency of HPTLC as a convenient, simple, and cost-effective technique for evaluating drug compatibility in a complex pharmaceutical matrix like TPN.

## Figures and Tables

**Table 1:** HPTLC method verification for analysing caffeine, ibuprofen, and midazolam.

Parameters	Caffeine	Ibuprofen	Midazolam
Selected mobile phase	Acetone: toluene: chloroform (4:3:3, *v*/*v*/*v*)	Toluene: methanol: ethyl acetate: glacial acetic acid (5.5:3.2:1:0.3, *v*/*v*/*v*/*v*)	Cyclohexane: toluene: diethylamine (7:1.5:1.5, *v*/*v/v*) with one drop 25% ammonia solution
Absorption maximum (λ_max_)	275 nm	271 nm	229 nm
R_F_ value	0.27 ± 0.03	0.67 ± 0.02	0.16 ± 0.03
Correlation coefficient (*R*^2^), average	0.9962	0.9970	0.9957
LOD, ng/band	1.95	252.22	10.82
LOQ, ng/band	5.90	764.30	32.78
Accuracy, average % recovery (range)	98.76–100.96	101.46–101.89	100.39–103.47
Intra-day precision, %RSD (range)	0.95–4.37	2.13–4.69	2.21–3.77
Inter-day precision, %RSD (range)	0.58–2.52	2.28–3.17	2.37–3.93
Repeatability, %RSD	2.00	2.98	2.82

**Table 2:** Physicochemical characteristics of caffeine, ibuprofen, and midazolam, and their respective salt forms [40,41].

Physicochemical Properties	Caffeine	Caffeine Citrate	Ibuprofen	Ibuprofen Arginine	Midazolam	Midazolam Hydrochloride
Water Solubility (mg/mL)	21.60	~40.00	0.021	>20.00	0.00987	9.06
logP	−0.07	−0.131	3.07	3.62	2.73	3.97
Characteristics	Near neutral (pKa ~ 0.60	Salt of weak base (pKa ~ 1.74)	Moderately acidic (pK_a_ ~ 4.50)	Salt of weak acid and basic amino acid (pK_a_ ~ 4.85)	Moderately basic (pK_a_ ~ 5.50)	Salt of weak base (pK_a_ ~ 6.00)

**Table 3:** Composition of 100 mL neonatal PN base solution and 25 mL pre-filled lipid emulsions.

KEMH-Preterm A	Each 100 mL Contains
PN Base Solution	
Glucose 25%	20.00 mL
Primene (IV amino acid solution)	27.00 mL
Water for injection	43.00 mL
Electrolytes	
Sodium	4.00 mmol
Potassium	2.00 mmol
Calcium	1.50 mmol
Phosphate	1.50 mmol
Magnesium	0.25 mmol
Acetate	2.00 mmol
Chloride	2.01 mmol
Additives	
Trace elements—Paediatric	0.74 mL
Heparin Sodium	50.00 Unit
**SMOF 20% Fat Emulsion with Vitamins**	**Each 25 mL contains**
SMOF fat emulsion	18.75 mL
Soluvit N Infant^®^ (Fresenius Kabi Australia Pty Limited, Mount Kuring-gai, NSW, Australia)	1.25 mL
Vitalipid Infant^®^ (Fresenius Kabi Australia Pty Limited, Mount Kuring-gai, NSW, Australia)	5.00 mL

**Table 4:** HPTLC instrumental and operating conditions for analysing caffeine, ibuprofen and midazolam.

HPTLC Instrumental and Operating Conditions	Caffeine	Ibuprofen	Midazolam
Software	visionCATS 4.0	visionCATS 4.0	visionCATS 4.0
Stationary phase	Silica gel 60 F254 HPTLC plates (20 × 10 cm)	Silica gel 60 F254 HPTLC plates (20 × 10 cm)	Silica gel 60 F254 HPTLC plates (20 × 10 cm)
Solvent	Methanol	Methanol	Methanol
Applied volume (µL)	2–10	1–5	2–7
Application rate (nLs^−1^)	80	150	80
Migration distance (cm)	8.0	8.5	7.0
Band width (mm)	8.0	8.0	8.0
Distance between two bands (mm)	11.4	11.4	11.4
Chamber saturation time (min)	20.0	15.0	30.0
Development time (min)	9	18	9
Temperature (°C) and Relative Humidity (%)	Room temperature (25 °C); 33%	Room temperature (25 °C); 33%	Room temperature (25 °C); 33%

## Data Availability

The original contributions presented in this study are included in the article/Supplementary Material. Further inquiries can be directed to the corresponding author.

## Supplementary Materials

The following supporting information can be downloaded at https://www.mdpi.com/article/10.3390/molecules31162904/s1. Figure S1: The absorption maxima (λ_max_) of sample bands with those of the corresponding reference standard bands of (a) caffeine, (b) ibuprofen, and (c) midazolam; Table S1: A paired Two-One-Sided Test (TOST) equivalence testing of drug concentrations (*n* = 3) of CAF, IBP and MDZ admixed in TPN over 24 h.
