# Supplementary material for: Exploring the Suitability of High-Performance Thin-Layer Chromatography for the Analysis of Drug Admixtures in Total Parenteral Nutrition Using Caffeine, Ibuprofen and Midazolam as Model Drugs
Source: Molecules. 2026 Aug 20;31(16):2904. doi: 10.3390/molecules31162904 (PMC13515485; doi:10.3390/molecules31162904)
Supplement: Supplementary file 1 [file molecules-31-02904-s001.zip › molecules-4496705-supplementary.pdf]

Supplementary Material

Exploring the suitability of High Performance Thin-Layer Chromatography for the analysis of drug admixtures in Total Parenteral Nutrition using caffeine, ibuprofen and midazolam as model drugs

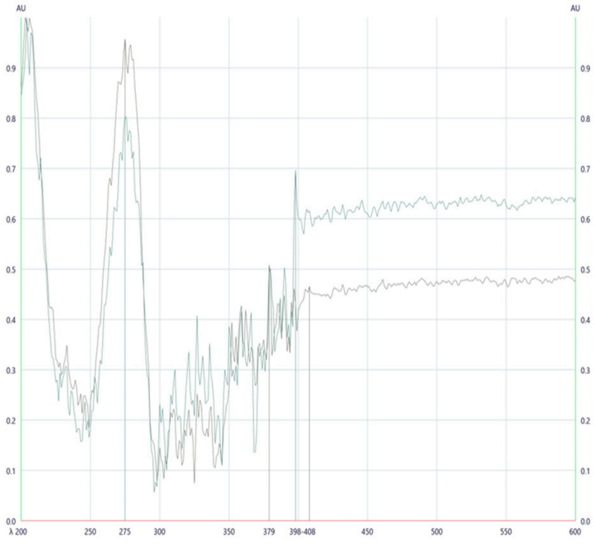

(a)

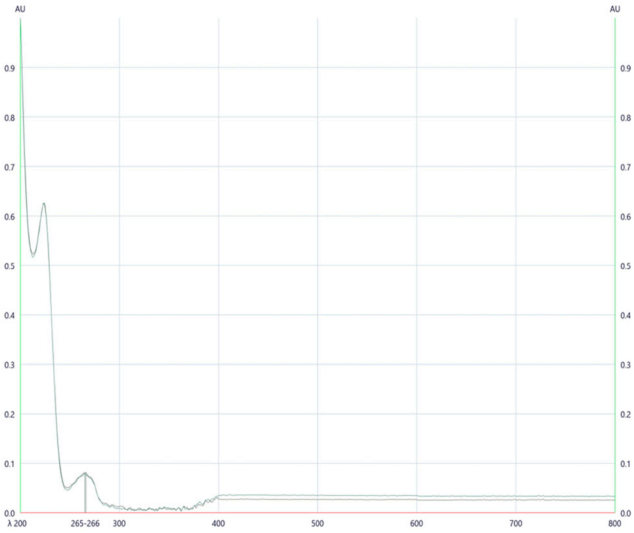

(b)

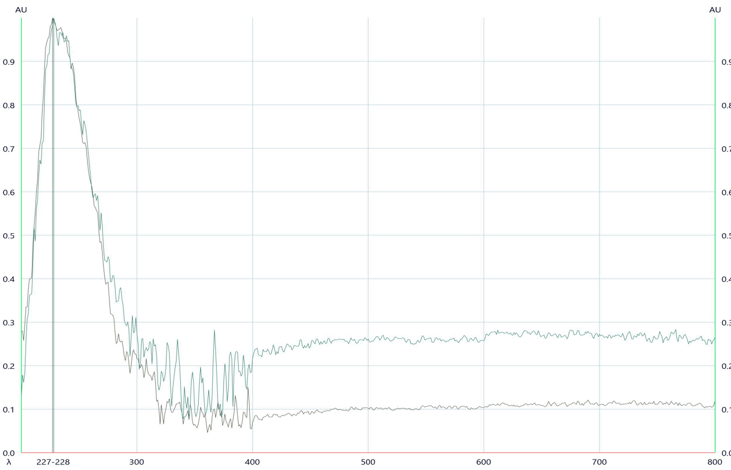

(c)

Figure S1: The absorption maxima (λ_{max}) of sample bands with those of the corresponding reference standard bands of (a) caffeine, (b) ibuprofen, and (c) midazolam.

Table S1. A paired Two-One-Sided Test (TOST) equivalence testing of drug concentrations (*n* = 3) of CAF, IBP and MDZ admixed in TPN over 24 h.

Comparison	TOST p-value (CAF)	TOST p-value (IBP)	TOST p-value (MDZ)
1 h vs 0 h	0.0016	0.0052	0.0243
2 h vs 0 h	0.0168	0.0228	0.0113
3 h vs 0 h	0.0023	0.0390	0.0317
4 h vs 0 h	0.0051	0.0237	0.0071
8 h vs 0 h	0.0177	0.0096	0.0179
24 h vs 0 h	0.0184	0.0545	0.0075
